# Either Non-Homologous Ends Joining or Homologous Recombination Is Required to Repair Double-Strand Breaks in the Genome of Macrophage-Internalized *Mycobacterium tuberculosis*

**DOI:** 10.1371/journal.pone.0092799

**Published:** 2014-03-21

**Authors:** Anna Brzostek, Izabela Szulc, Magdalena Klink, Marta Brzezinska, Zofia Sulowska, Jaroslaw Dziadek

**Affiliations:** Institute of Medical Biology, Polish Academy of Sciences, Lodz, Poland; University of Massachusetts Medical School, United States of America

## Abstract

The intracellular pathogen *Mycobacterium tuberculosis* (Mtb) is constantly exposed to a multitude of hostile conditions and is confronted by a variety of potentially DNA-damaging assaults *in vivo*, primarily from host-generated antimicrobial toxic radicals. Exposure to reactive nitrogen species and/or reactive oxygen species causes different types of DNA damage, including oxidation, depurination, methylation and deamination, that can result in single- or double-strand breaks (DSBs). These breaks affect the integrity of the whole genome and, when left unrepaired, can lead to cell death. Here, we investigated the role of the DSB repair pathways, homologous recombination (HR) and non-homologous ends joining (NHEJ), in the survival of Mtb inside macrophages. To this end, we constructed Mtb strains defective for HR (Δ*recA*), NHEJ [Δ(*ku,ligD*)], or both DSB repair systems [Δ(*ku,ligD,recA*)]. Experiments using these strains revealed that either HR or NHEJ is sufficient for the survival and propagation of tubercle bacilli inside macrophages. Inhibition of nitric oxide or superoxide anion production with L-NIL or apocynin, respectively, enabled the Δ(*ku,ligD,recA*) mutant strain lacking both systems to survive intracellularly. Complementation of the Δ(*ku,ligD,recA*) mutant with an intact *recA* or *ku-ligD* rescued the ability of Mtb to propagate inside macrophages.

## Introduction

Macrophages (MØs), which are derived from monocytes, are professional phagocytic cells specialized in ingesting and killing pathogens. The antimicrobial activity of MØs is due, in part, to the generation of large amounts of highly toxic molecules, including reactive oxygen species (ROS), such as superoxide anion (•O_2_^-^), hydrogen peroxide (H_2_O_2_), hydroxyl radicals (•OH) and hydroxyl anion (OH^-^), as well as reactive nitrogen species (RNS), such as nitric oxide (NO) and peroxynitrite anion (ONOO^-^). These reactive species cause oxidative damage to a wide variety of targets, including DNA. The accumulation of DNA damage in the form of oxidation, depurination, methylation, and deamination can cause single- and double-strand breaks (DSBs) that affect the integrity of the whole genome; when left unrepaired, these breaks can lead to cell death [1], [2]. The major DSB repair pathway in bacteria is homologous recombination (HR), which promotes strand exchange between DNA molecules, with RecA acting as a key protein. During HR, a complex of single-stranded DNA coated by RecA protein recognizes homology in double-stranded DNA and invades it, subsequently catalyzing strand exchange [3], [4]. We and others have shown that, in addition to HR, mycobacteria possess a prototypical non-homologous ends joining (NHEJ) apparatus encoded by evolutionarily conserved *ku* and *ligD* genes [5]–[9], as well as a single-strand annealing (SSA) pathway [10]. In the NHEJ process, Ku protein binds to the DNA ends and subsequently interacts with multifunctional LigD, which covalently joins together broken DNA strands [11]. Both HR and NHEJ systems have complementary roles in repairing DSBs, but act independently [12], [13].

*Mycobacterium tuberculosis* (Mtb) is expected to sustain a variety of potentially DNA-damaging assaults *in vivo* [14]. In the very early stage of infection, outside the host cell, mycobacteria might be exposed to desiccation, which is a physiological equivalent of ionizing radiation (IR) [15]. As is the case for IR, the cytotoxicity of desiccation derives from the formation of DSBs, which are also caused by a variety of endogenous and exogenous agents [16], [17]. In the host, mycobacterial DNA is a biological target for RNS and ROS, which can damage lipids, proteins and nucleic acids; in the case of DNA, the interaction with these toxic radicals is mutagenic. DNA integrity can also be affected indirectly by damage to cellular components required for protection or propagation of DNA [18]. It has been postulated that there is a switch between aerobic and anaerobic metabolism in the granuloma formation process. Additional endogenous reactive species are also likely to be generated by this switch and from the partial reduction of terminal electron acceptors during respiration [18].

The roles of HR and NHEJ in repairing DNA damage in mycobacteria exposed to ultraviolet radiation (UV), IR, desiccation, methyl methanesulfonate (MMS) or mitomycin C and in respect with stability of repeated sequences able to form non-B DNA structures have been extensively studied *in vitro* [7]–[9], [19]. To date, however, the significance of HR and NHEJ during the Mtb infection process has not been investigated.

Here, we addressed the role of DSB repair systems in Mtb infection by first engineering an HR-defective Mtb mutant lacking a functional copy of the *recA* gene (Δ*recA*), an NHEJ-defective mutant lacking Ku and ligase D proteins [Δ(*ku,ligD*)], and an HR/NHEJ-defective mutant lacking all these genes [Δ(*ku,ligD,recA*)]. Using these mutant strains, we then assessed the requirement of HR and/or NHEJ for tubercle bacilli survival inside human MØs.

## Materials and Methods

### Reagents

RPMI-1640 culture medium and Hanks' balanced salt solution (HBSS) were obtained from Gibco (Inchinnan, Scotland). Fetal bovine serum (FBS) and human type AB serum were purchased from PAA The Cell Culture Company (Pasching, Austria). Middlebrook 7H10 agar, Middlebrook 7H9 broth, and Middlebrook OADC enrichment were acquired from Becton Dickinson (Franklin Lakes, NJ, USA). Phorbol-12-myristate-13-acetate (PMA), diethylenetriamine/nitric oxide adduct (DETA/NO), menadione, 4′-hydroxy-3′-methoxyacetophenone (apocynin), L-N^6^-(1-iminoethyl)lysine dihydrochloride (L-NIL), Triton X-100, β-mercaptoethanol, penicillin (10,000 U/ml)/streptomycin (10 mg/ml) solution (P/S), fluorescein isothiocyanate (FITC), Tween-80, 37% formaldehyde (FA) solution, and bovine serum albumin (BSA) were purchased from Sigma-Aldrich (St. Louis, MO, USA).

### Gene replacement constructs and gene disruption

**Figure 1:**
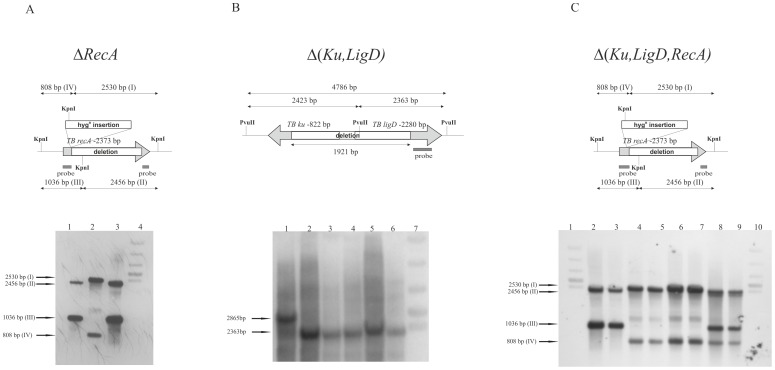
Confirmation of obtained mutants by Southern blotting. (A) Replacement of wild-type *recA* with its mutated copy, Δ*recA*. The chromosomal DNA was digested with *Kpn*I. The hybridization probe was amplified based on pMG 22 carrying Δ*recA* gene as a template, and contained 5′(377 bp) and 3′(75 bp) fragments of *recA*. The wild-type and mutant strains revealed a different hybridization patterns: I- 2530 bp band representing 3′ KpnI Δ*recA* (629 bp, carrying 75 bp of 3′*recA* end and 554 bp downstream region joined to *hyg* ^R^ cassette fragment of 1901 bp); II- 2456 bp band representing 3′KpnI *recA* wild type carrying 1666 bp of 3′*recA* gene and 790 bp of downstream region; III- 1036 bp band representing 5′KpnI *recA* wild type carrying 707 bp of 5′ *recA* gene and 329 bp of upstream region; IV- 808 bp band representing 5′KpnI Δ*recA* (706 bp, carrying 377 bp of 5′ *recA* gene fragment and 329 bp of upstreamregion joined to *hyg* ^R^ cassette fragment of 102 bp), Lanes: 1, Mtb wild-type (control); 2, DCO Δ*recA* (double-crossover homologous-recombination mutant); 3, SCO Δ*recA-recA* (single-crossover homologous-recombination mutant); 4, 1-kb marker. (B) Replacement of wild-type *ku* and *ligD* genes with their mutated copies, Δ*ku* and Δ*ligD*. The chromosomal DNA was digested with *Pvu*II. The 3′ end of the *ligD* gene was used as a hybridization probe. Lanes: 1, DCO Δ(*ku,ligD*); 2–5, DCO wild-type *ku,ligD*; 6, Mtb wild-type (control); 7, 1-kb marker. (C) Replacement of wild-type *recA* with its mutated copy (Δ*recA*) in a strain carrying inactivated *ku* and *ligD* genes. The chromosomal DNA was digested with *Kpn*I. The 3′ end of *recA* was used as a hybridization probe. The probe was amplified based on pMG 22 carrying Δ*recA* gene, and contained 5′ (377 bp) and 3′ (75 bp) fragments of *recA*. The wild-type and mutant strains revealed a different hybridization patterns: I- 2530 bp band representing 3′ KpnI Δ*recA* (629 bp, carrying 75 bp of 3′*recA* end and 554 bp downstream region joined to *hyg* ^R^ cassette fragment of 1901 bp); II- 2456 bp band representing 3′KpnI *recA* wild type carrying 1666 bp of 3′*recA* gene and 790 bp of downstream region; III- 1036 bp band representing 5′KpnI *recA* wild type carrying 707 bp of 5′ *recA* gene and 329 bp of upstream region; IV- 808 bp band representing 5′KpnI Δ*recA* (706 bp, carrying 377 bp of 5′ *recA* gene fragment and 329 bp of upstream region joined to *hyg* ^R^ cassette fragment of 102 bp). Lanes: 1 and 10, 1-kb marker; 2 and 3, Mtb wild-type (control); 4–7, DCO Δ(*ku,ligD,recA*); 8 and 9, SCO Δ(*ku,ligD,recA*)-*recA*.

To perform unmarked deletions in the *recA* (Rv2737c) gene of *Mtb*, suicidal recombination delivery vector was constructed. The recombination vector contained the 5′ end of the *recA* gene (377 bp) with upstream region connected to the 3′ end of the gene (72 bp) with downstream region. The 5′ and 3′ fragments of the gene were ligated out of frame, resulting in expression of a non-functional protein. To perform unmarked deletion in the *ligD* (Rv0938) and *ku* (Rv0937c) genes the recombination vector carrying the 3′ end of *ku* (433 bp) with downstream region connected to the 3′ end of *ligD* (901 bp) with downstream region was constructed. The protocol of [20] was used to disrupt *ligD, ku*, and *recA* at their native chromosomal loci. Plasmid DNAs (pAB215, pMG22) were treated with NaOH (0.2 mM) and integrated into the *M. tuberculosis* H37Rv chromosome by homologous recombination, as described previously [21]–[24]. The resulting single-crossover (SCO) colonies were blue, kanamycin resistant, and sensitive to sucrose (2%). The site of recombination was confirmed by polymerase chain reaction (PCR) and Southern hybridization. SCO strains were further processed to select for double-crossover (DCO) mutants, which were white, kanamycin sensitive, and resistant to sucrose. PCR and Southern hybridization were used to distinguish between wild-type and DCO mutant colonies. The probes were generated by PCR and labeled using a nonradioactive primer extension system (DIG-labeling system; Amersham, Sweden) (Fig. 1). The mutant strains were constructed by subsequent replacement of endogenous *ku-ligD* and *recA* genes. Complementation vectors were engineered by amplifying the *ligD-ku* genes with their putative promoter of Mtb with Ku-DTbXb and Ku-DTbHi primers and the *recA* gene and it's putative promoter of *Mycobacterium smegmatis* with MsrecAr and MsrecAPs primers from genomic DNA and cloning them into XbaI/HindIII (*ligD-ku*) or XbaI/EcoRI (*recA*) restriction sites of pMV306Km integration vector. The final constructs were verified by sequencing analysis. All plasmids, strains, and primers used in this work are listed in Table S1.

### Bacterial strains and growth conditions

Bacteria for infections were prepared by growing wild-type and mutants strains in Middlebrook 7H9 broth containing 10% OADC enrichment and 0.05% Tween-80 (in roller bottles) until they reached an optical density at 600 nm (OD_600_) of 1 (4–6 days). A portion of the bacterial culture was suspended in Middlebrook 7H9 broth (∼1×10^9^ bacilli/ml) and labeled with 100 μg/ml of FITC for 2 hours at room temperature in the dark with gentle agitation. FITC-labeled bacteria were washed once with Middlebrook 7H9 broth supplemented with 4% BSA and then twice with Middlebrook 7H9 broth without BSA. Unlabeled and FITC-labeled bacteria were divided into equal portions and stored at −85°C. After 1 week, one portion of unlabeled and one portion of FITC-labeled bacteria were thawed and the number of bacteria was determined by measuring colony-forming units (CFUs). FITC-labeled bacteria were used in experiments determining bacterial uptake only. Before infection bacteria were thawed, washed twice in RPMI-1640 medium, and opsonized (or not) by incubating with 30% human type AB serum for 30 minutes at 37°C with gentle agitation. Bacteria were then washed once with RPMI-1640 and suspended in culture medium (CM, see below) without P/S. After disrupting clumps by multiple passages through a 25-gauge needle, Mtb were serially diluted in CM without P/S.

### Growth of Mtb strains after exposure to UV radiation

Mtb strains were cultured in Middlebrook 7H9 supplemented with 10% OADC. A sample of each strain was taken during exponential and stationary phases of Mtb growth, and serial dilutions were prepared and plated on Middlebrook 7H10 agar supplemented with 10% OADC. Next, plates were exposed (or not) to UV radiation at doses of 5, 10, or 15 mJ/cm^2^ at room temperature in the dark. Afterwards, bacteria were incubated for 21 days at 37°C protected from light. After this incubation period, the number of colonies was counted.

### Growth of Mtb strains in the presence of NO or •O_2_^-^ *in vitro*

On the day of the experiment, an inoculum of each Mtb strain (wild-type and Δ(*ku,ligD,recA*), Δ(*ku,ligD*) and Δ*recA* mutants) was added into fresh 7H9 Middlebrook medium containing 10% OADC and grown to a final OD_600_ of 0.1. Then, the NO donor diethylenetriamine/NO (25, 50, 100, 500 or 1000 μM) or the •O_2_^-^ donor menadione (10, 20, 40, 50 and 100 μM) was added (or not) and bacteria were incubated for 6 days at 37°C. On days 1, 4 and 6 after exposure, OD_600_ values of all cultures were measured using a BioPhotometer Plus (Eppendorf, Hamburg, Germany); on day 6, bacteria were plated on Middlebrook 7H10 agar supplemented with 10% OADC. After 21 days of incubation (37°C), the number of CFUs was counted.

### Cell line

The human monocyte-macrophage cell line THP-1 (ATCC TIB-202; American Type Culture Collection, Manassas, VA, USA) was maintained in culture medium (CM) containing RPMI-1640 medium supplemented with 1 mM sodium pyruvate, 10% FBS, 0.05 mM β-mercaptoethanol, and antibiotics (100 U/ml of penicillin and 100 μg/ml of streptomycin). Cells were passaged every 3 days.

Undifferentiated THP-1 monocytes (minimum eighth passage) were differentiated into MØs by incubating with 20 ng/ml of PMA for 24 hours (37°C, 5% CO_2_) in CM without antibiotics. The ability of MØs to attach to the plastic surface of plates was confirmed by light microscopy. The expression of specific surface molecules, such as CD14, TLR2 (Toll-like receptor 2) and CR3 (complement receptor 3), on MØs, indicative of THP-1 differentiation, was determined as described previously [25]. After differentiation MØs were washed once with RPMI-1640 medium and suspended in CM without antibiotics.

### Phagocytosis and intracellular growth of Mtb strains

Phagocytosis was conducted in 8-well Permanox chamber slides (Nunc, Roskilde, Denmark). Obtained after THP-1 differentiation MØs (1×10^5^ cells/well) were infected with FITC-labeled Mtb strains (1×10^6^ Mtb/well) at a multiplicity of infection (MOI) of 10 in CM without antibiotics. After 2 hours of infection, MØs were extensively washed three times with HBSS and then MØs with ingested bacteria were fixed by incubating with 3% FA for 15 minutes (37°C, 5% CO_2_) and then washed twice with HBSS. The number of infected MØs and the number of bacteria engulfed by one MØ were estimated by fluorescence microscopic examination (Nikon ECLIPSE TE 2000 U, Nikon, Tokyo, Japan). In each case, 200 MØs were counted.

Intracellular growth of bacteria was estimated using the CFU method. Obtained after THP-1 differentiation, MØs (1×10^5^ cells/well in a 24-well plate) were first treated with L-NIL (25 μM) or apocynin (50 μM) for 30 minutes, or left untreated. Next, MØs were infected with opsonized or non-opsonized unlabeled Mtb strains at an MOI of 1 in culture medium without antibiotics. After 2 hours of infection, non-ingested Mtb were removed by extensive washing MØs three times with HBSS and after that the culture medium containing 1 mg/ml of gentamycin was added for 2 hours to kill extracellular bacteria, and then MØs were washed twice with HBSS. Thereafter, fresh CM without antibiotics was added and MØs with ingested bacteria were further cultured. On days 0, 2, 4 and 6, MØs were lysed with 1 ml of 0.2% Triton X-100. Next, lysates diluted in HBSS were plated on Middlebrook 7H10 agar supplemented with 10% OADC. After 21 days of incubation (37°C), the number of colonies was counted. Data are presented as fold-increase in CFU, calculated as CFUs on day 6/CFUs on day 0.

To confirm that apocynin inhibits the ROS production in MØs we used luminol enhanced chemiluminescence method (CL). MØs (1×10^5^ cells/well) were incubated (37°C; 5% CO_2_) with various concentration of apocynin (10, 25, 50 μM) for 30 minutes or left untreated. Afterwards, PMA (1 μg/ml) to initiate ROS production, luminol (1 mM) and horseradish peroxidase (40 U) to enhance CL were added to MØs. Emitted light was measured at 37°C, 4 hours with 5 minutes interval, using fluoroscan Ascent FL (Labsystems, Helsinki, Finland). Data were acquired as relative light units (RLU).

### Statistical analysis

All data are presented as means ± SEMs and were analyzed with non-parametric Mann-Whitney *U* test using Statistica 10.0 software for Windows. Statistical significance was defined as p≤0.05.

## Results

The RecA, Ku, and LigD proteins are essential participants in HR and NHEJ processes, both of which are involved in the repair of DSBs in mycobacteria [5]–[7], [9], [26]. A gene-replacement strategy was used to inactivate *recA*, *ku-ligD*, or all three genes in the Mtb H37Rv strain. The resultant mutants were than examined for growth under conditions that promote DNA damage *in vitro* and *in vivo*.

### The repair of UV radiation-induced DNA damage in Mtb requires intact RecA

**Figure 2:**
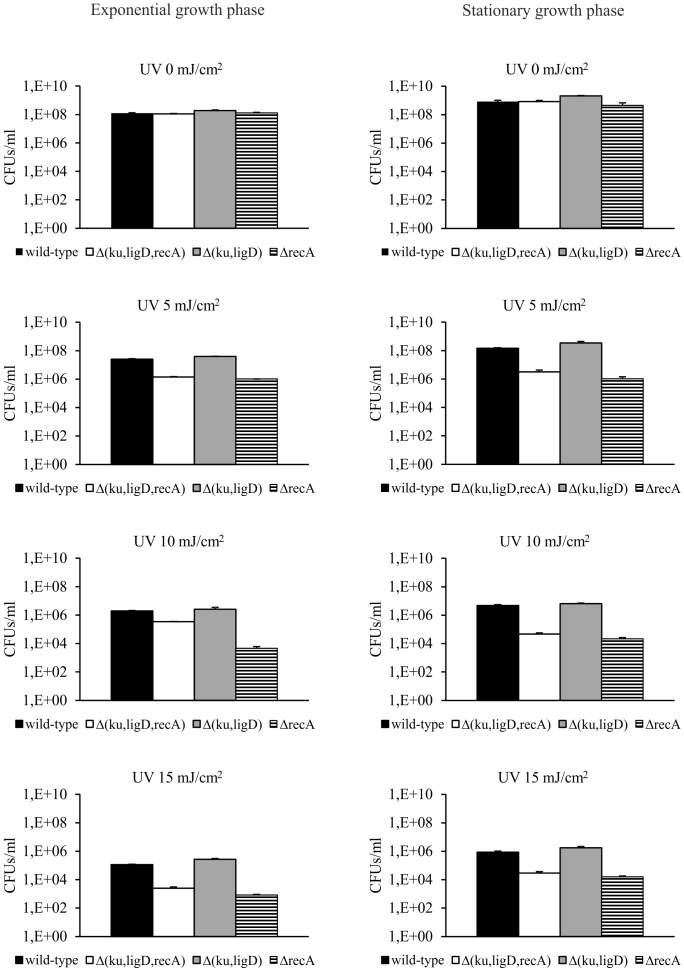
Survival of Mtb strains after UV radiation. Mtb strains were exposed to the indicated doses of UV light during exponential and stationary growth phases. Following irradiation, the number of viable bacteria was determined by the CFU method. Data are presented as CFUs/ml, expressed as means ± SEM (n = 3).

It is well known that bacteria defective for RecA synthesis are hypersensitive to UV radiation [8], [27]. The *Mtb* mutants, Δ(*ku,ligD,recA*), Δ(*ku,ligD*), and Δ*recA* generated in this work were examined after exposure to UV radiation *in vitro*. As expected, strains lacking an intact *recA* gene [Δ(*ku,ligD,recA*) and Δ*recA*] were much more sensitive to UV exposure than were wild-type or Δ(*ku,ligD*) strains (Fig. 2). In contrast, the UV-sensitivity of the NHEJ mutant, Δ(*ku,ligD*), was not significantly different from that of the wild-type strain in exponential or stationary growth phases (Fig. 2). Collectively, the results of this *in vitro* analysis confirm the UV-hypersensitive phenotype of Mtb mutants lacking an intact *recA* gene and demonstrate that intact RecA, but not NHEJ, is required to repair DNA damage caused by UV stress.

**Figure 3:**
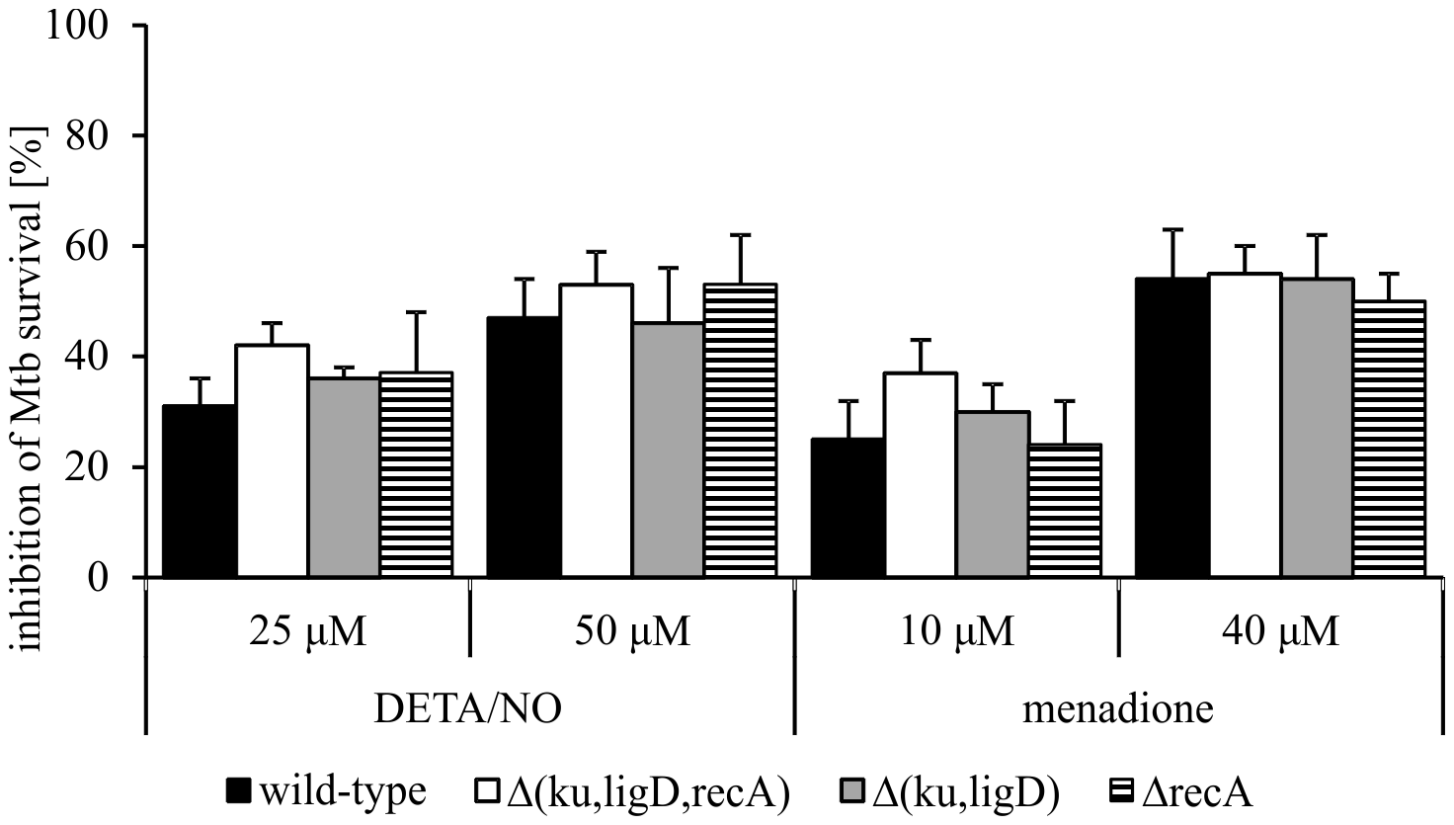
Survival of Mtb strains in the presence of DETA/NO or menadione. Mtb strains were cultured in 7H9 Middlebrook with 10% of OADC, with or without various concentrations of DETA/NO or menadione for 6 days. The number of viable bacteria was determined by the CFU method. Data are presented as percentage of Mtb survival inhibition, expressed as means ± SEM (n = 3).

NO, •O_2_^-^, and ONOO^-^ are known to cause oxidative damage to a number of molecules, including DNA [28]. The accumulation of such DNA damage may result in single- and double-strand brakes. The wild-type Mtb strain and Δ*recA*, Δ(*ku,ligD*), and Δ(*ku,ligD,recA*) mutants were exposed to a range of concentrations (25–1000 μM) of the slow-release NO donor, DETA/NO. The OD_600_ of cultures was determined on days 0, 1, 4 and 6, and CFUs were further assessed on culture day 6. DETA/NO at a concentration of 100 μM was lethal for all tested strains. At lower concentrations (25 and 50 μM), DETA/NO similarly inhibited the survival of all tested strains (Fig. 3). At 50 μM, DETA/NO inhibited survival of wild-type Mtb by 47%±4% and that of its mutants Δ(*ku,ligD,recA*), Δ(*ku,ligD*), and Δ*recA* by 53%±6%, 46%±6%, 53%±5%, respectively.

Next, wild-type and Mtb mutant strains were exposed to the •O_2_^-^ donor menadione at concentrations ranging from 10 to 100 μM. Menadione was lethal to all tested Mtb strains at concentrations of 50 and 100 μM. Similar to DETA/NO, menadione at a concentration of 40 μM reduced the survival of all Mtb strains to a similar degree: wild-type, 54%±11%; Δ(*ku,ligD,recA*), 56%±9%; Δ(*ku,ligD*), 54%±8%; and Δ*recA*, 50%±5% (Fig. 3).

### Either NHEJ or HR is required for survival of Mtb inside MØs

**Figure 4:**
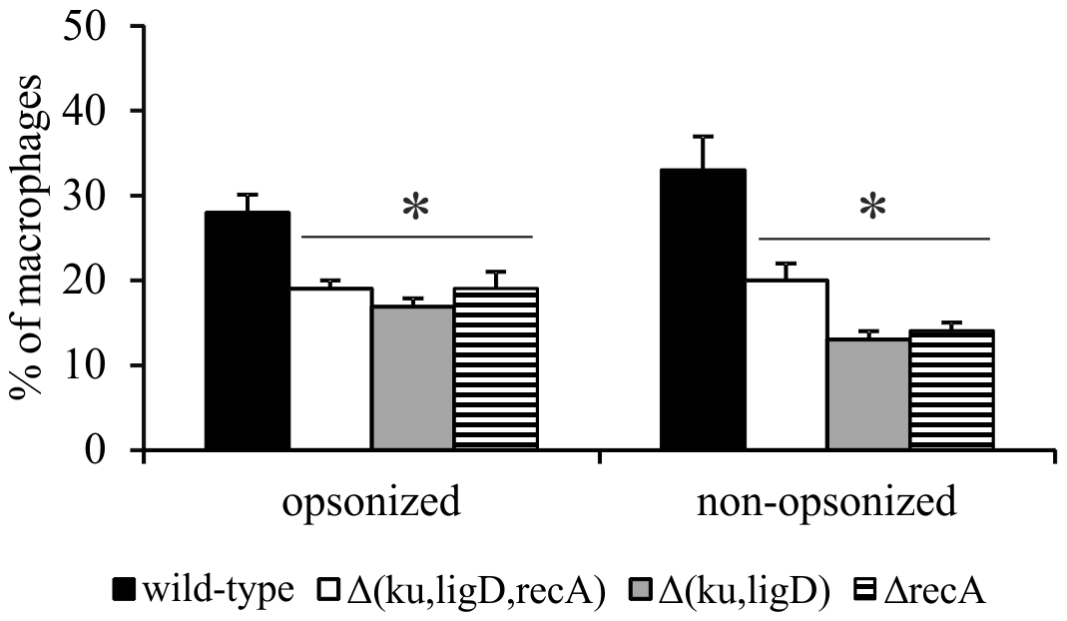
The percentage of MØs engaged in the ingestion of Mtb strains. MØs were infected with FITC-labeled wild-type Mtb or its mutants, Δ(*ku,ligD,recA*), Δ(*ku,ligD*) or Δ*recA*, for 2 hours (MOI  = 1) and washed with HBSS. The percentages of MØs infected with Mtb strains were calculated according to the formula, MØ with bacteria ×100/number of counted MØs, and expressed as means ± SEM (n = 5; *p≤0.04, Δ(*ku,ligD,recA*), Δ(*ku,ligD*), and Δ*recA* vs. wild-type; Mann-Whitney *U* test).

In order to determine the infection efficiencies of mutants relative to that of the wild-type strain, we estimated the percentage of MØs infected with bacteria. The percentage of MØs engaged in the ingestion of Δ(*ku,ligD,recA*), Δ(*ku,ligD*), or Δ*recA* strains (10–20%) was significantly lower than that for wild-type Mtb (30–40%) (Fig. 4).

**Figure 5:**
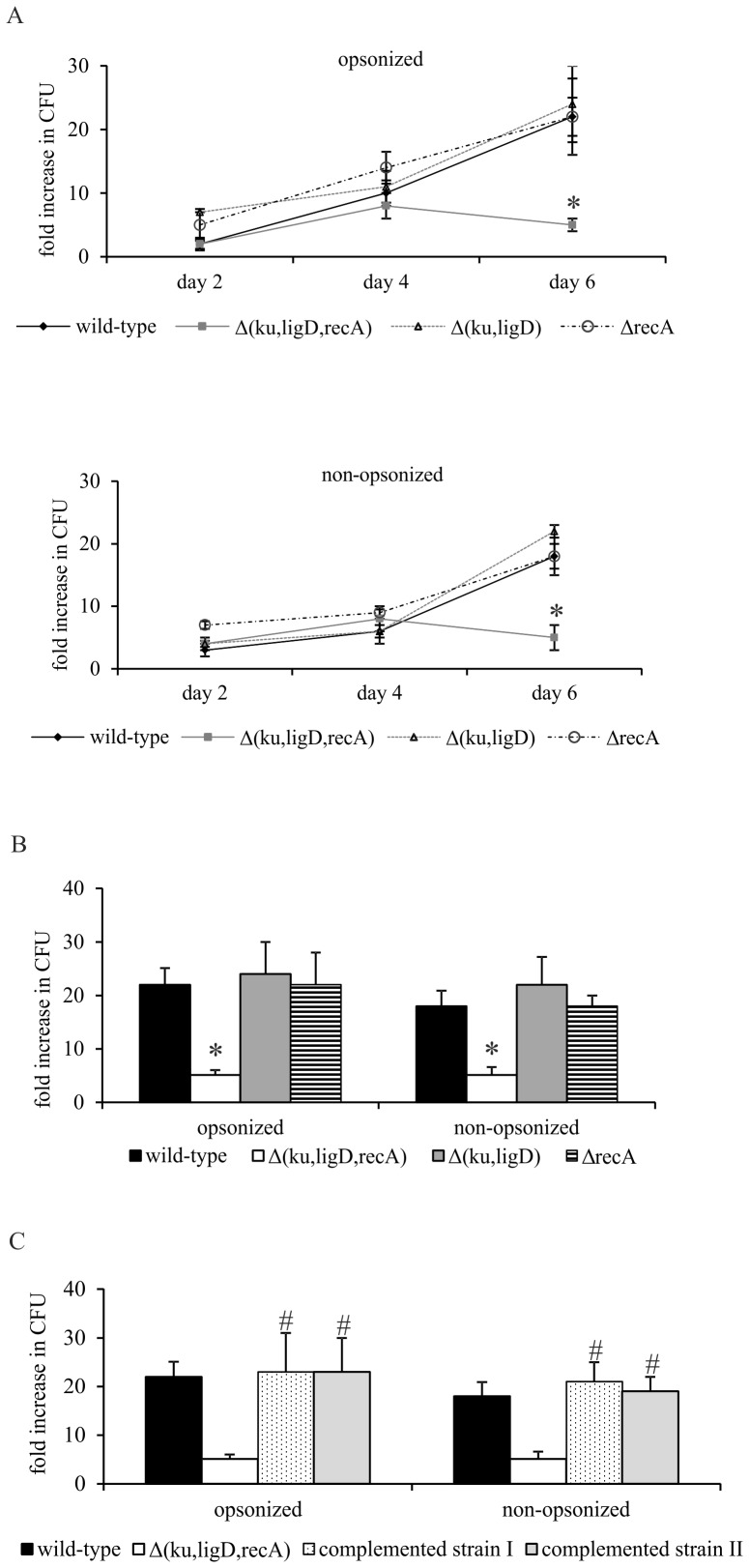
Survival of Mtb strains inside MØs. (A) MØs were infected with Mtb wild-type or its mutants, Δ(*ku,ligD,recA*), Δ(*ku,ligD*) or Δ*recA*, for 2 hours (MOI  = 10), washed with HBSS, and cultured for 2, 4, and 6 days. (B and C) MØs were infected with Mtb wild-type or its mutants (B) or complemented strains Δ(*ku,ligD,recA*)-*P_recA_recA* – complemented strain I or Δ(*ku,ligD,recA*)-*P_kuligD_ku-ligD* – complemented strain II (C) for 6 days. On the day of infection and after 6 days of culture, MØs were lysed with Triton X-100 and cell lysates were plated onto Middlebrook 7H10 agar supplemented with 10% OADC. After 21 days of culture, CFUs were counted. Data are presented as fold increase in CFUs, expressed as means ± SEM (n = 5; *p≤0.05, Δ(*ku,ligD,recA*) vs. wild-type; #p≤0.05, complemented strain I and II vs. Δ(*ku,ligD,recA*) Mann-Whitney *U* test).

Next, the survival of wild-type and Δ(*ku,ligD,recA*), Δ(*ku,ligD*), and Δ*recA* Mtb strains was determined 2, 4, and 6 day post-infection. A similar fold-increase in CFUs for all strains, opsonized and non-opsonized, was observed until day 4. However, on day 6, the fold-increase in CFUs for Δ(*ku,ligD,recA*) was considerably diminished compared with that of the other strains tested (Fig. 5A). Based on these observations, we compared the intracellular replication of Mtb strains on day 6 post-infection. We found that survival was similar for wild-type, Δ(*ku,ligD*) and Δ*recA* strains, both opsonized and non-opsonized. Only the growth of the triple mutant, Δ(*ku,ligD,recA*), was highly and significantly attenuated in MØs compared to that of the wild-type Mtb and other mutants (Fig. 5B). This observation was verified using the *recA-* or *ku-ligD-* complemented strains Δ(*ku,ligD,recA*)-*P_recA_recA* and Δ(*ku,ligD,recA*)-*P_kuligD_ku-ligD*, respectively. Either complementation with RecA or co-expression of both Ku and LigD attenuated the phenotype of the Δ(*ku,ligD,recA*) triple mutant, confirming that survival of tubercle bacilli inside MØs can be supported by either DSB repair pathway (Fig. 5C).

**Figure 6:**
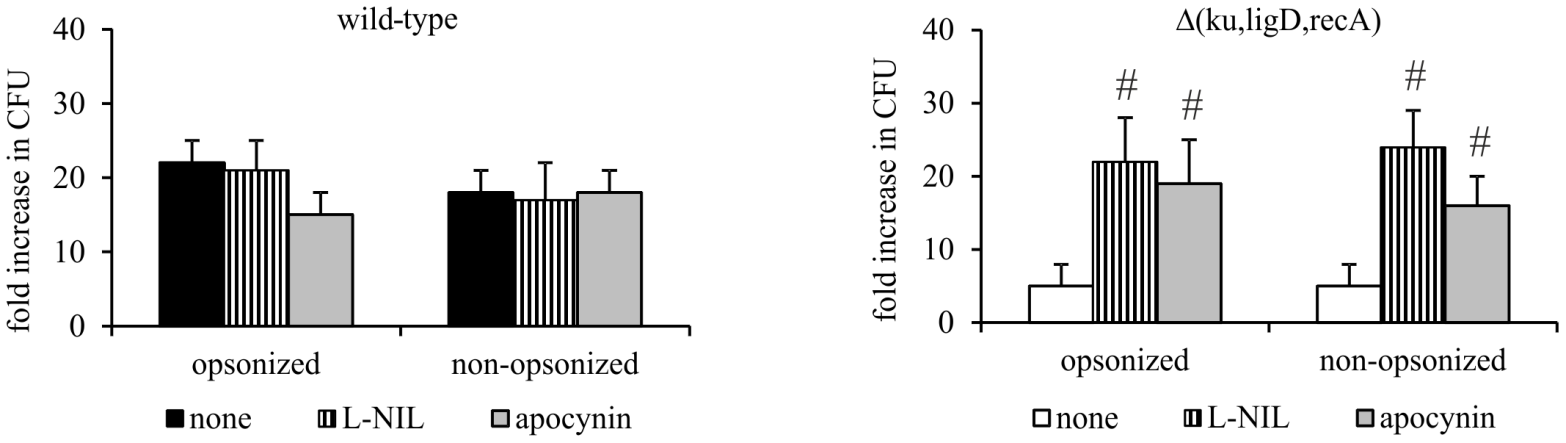
Effect of inhibition of ROS and NO production on the survival of Mtb strains inside MØs. MØs were incubated with apocynin or L-NIL for 30 minutes, then infected with Mtb wild-type or Δ(*ku,ligD,recA*) for 2 hours (MOI  = 10) and washed with HBSS. On the day of infection and after 6 days of culture, MØs were lysed with Triton X-100 and cell lysates were plated onto Middlebrook 7H10 agar supplemented with 10% OADC. After 21 days of culture, CFUs were counted. Data are presented as fold increase in CFUs, expressed as means ± SEM (n = 5; #p≤0.04, Δ(*ku,ligD,recA*) vs. Δ(*ku,ligD,recA*) + apocynin or L-NIL; Mann-Whitney *U* test).

These results led us to question which reactive species — NO or •O_2_^-^ — participated in the intracellular killing of the Δ(*ku,ligD,recA*) mutant. To address this question, we incubated MØs with L-NIL or apocynin to inhibit the production of NO or •O_2_^-^ by MØs, respectively prior to infection with Mtb. These experiments revealed that inhibition of NO or •O_2_^-^ production strongly enhanced the intracellular survival of the triple mutant, possibly indicating that both toxic species contribute to the decreased viability of the Δ(*ku,ligD,recA*) mutant (Fig. 6). Control experiments showed that apocynin inhibited ROS production by PMA-stimulated MØs, decreasing the amount of fluorescently detected ROS (expressed as relative light units [RLU]) from 348±62 to 116±12; by comparison, RLU values for unstimulated MØs were 74±1.

## Discussion

Numerous pathways operate to repair DNA damage in bacteria. Notably, damage repair pathways are essential for the virulence and survival of other intracellular pathogens like *Neisseria meningitides*, *Coxiella burnetii*, and *Vibrio cholerae* [29]–[31]. In most bacterial systems, adaptation to environmental stress is predicated on the activity of SOS-inducible, error-prone repair polymerases of the Y polymerase superfamily. Their predicted physiological roles are fulfilled in *M. tuberculosis* by the damage-inducible C family polymerase, DnaE2, which is responsible for damage-induced base substitution mutagenesis. Significantly, deletion of *dnaE2* results in damage hypersensitivity and is associated with attenuation of the late stage of infection in a murine model [32]. In addition, several base-excision-repair enzymes have also been identified and shown to be required for *in vivo*, but not *in vitro*, growth of *M. tuberculosis* [33]. Moreover, mutations in *uvrD1*, thought to be part of the nucleotide-excision-repair pathway (NER) as well as replication restart and recombination, result in RNS susceptibility *in vitro* and reduced capacity to resist ROS and RNS *in vivo* [34]. Elimination of *uvrD1* significantly affects the chronic stage of infection and impacts the ability of Mtb to replicate and persist in a mouse model of infection [35]. On the other hand, Mtb defective for UvrA, mediating the initial step of NER, are not attenuated in MØs, suggesting that NER is not required under these conditions [35]. Mtb defective for UvrB, the other NER component, exhibit a slightly reduced ability to survive in bone marrow-derived MØs and show modestly attenuated infection in mice [34] as well as in primate lungs [36].

Little is known about the role of DSB repair pathways during infection. A *recA* mutant of the *M. bovis* BCG strain causes no detectable phenotype in mice for up to 80 days after infection [27], possibly indicating that nitrosative or oxidative stresses do not induce cytotoxic DNA damage in the murine model. However, the possibility that the attenuated phenotype of BCG may have masked a *recA* deficiency cannot be excluded. The attenuation of *recA* mutants during infection has been described for other bacteria, including *Burkholderia spp*. [37], [38], *Salmonella enterica* [39] and *Acinetobacter baumannii* [40], but not *Porphyromonas gingivalis* [41]. In our study, the *recA* mutant appeared to be sensitive to the DNA-damaging agent UV *in vitro*, but was not attenuated in THP-1-derived MØs. Inactivation of NHEJ did not result in a detectable phenotype either *in vitro* or inside MØs. It was previously found that the absence of NHEJ sensitizes fast-growing mycobacteria to desiccation and IR during the stationary phase of growth [9], but not to mitomycin C [7] or UV [8]. In the current study, Mtb were only clearly attenuated in MØs if both DSB repair pathways were disrupted. Moreover, virulence was restored by complementation of either pathway. We conclude that intracellular exposure of Mtb to RNS and ROS results in the formation of DSBs, which must be repaired by HR or NHEJ—notwithstanding the fact that Mtb expression profiles suggest that HR genes, but not *ligD* and *ku*, are regulated during infection [14], [42]–[44]. NHEJ may nevertheless be important for Mtb survival within MØs, where they are continually exposed to the genotoxic defense mechanisms of host cells. Moreover, in some cells it become an only DSB repair pathway available during latency or reactivation from latency in cases where no daughter chromatid for HR is present [45].

MØs infected with Mtb generate various oxygen species, including •O_2_^-^ and NO, which are capable of damaging microbial DNA. Their significance in the host defense against Mtb is well documented [46], [47]. However, it has been reported that ONOO^-^, a product of •O_2_^-^ and NO interaction, plays a more important role in the killing of intracellular pathogens like Mtb than do •O_2_^-^ or NO themselves [2]. Using inhibitors of •O_2_^-^ or NO production we found that inhibition of intracellular replication of the Mtb Δ(*ku,ligD,recA*) mutant in MØs was dependent on the presence of both •O_2_^-^ and NO, suggesting that ONOO^-^ is required for effective induction of DSBs and killing of mutants defective in both NHEJ and HR. These observations are also supported by our *in vitro* studies demonstrating that neither NO nor •O_2_^-^ donors themselves induced DSBs in Mtb. Peroxinitrite anion is responsible for: inactivation of enzymes containing Fe-S clusters, S-nitrosilation of thiols, tyrosine nitration, modification of deoxyribonucleotides, interaction with DNA repair system, and DNA strand breaking what consequently leads to bacteria's death [48], [49].

We also observed a lower uptake of all mutants by MØs compared to wild-type Mtb. This might suggest that inactivation of either DSB repair pathway disrupts the metabolism of Mtb, resulting in modifications to the cell envelope. This phenomena did not further influence the fate of Mtb mutants engulfed by THP-1-derived MØs since only the Δ(*ku,ligD,recA*) strain was unable to propagate inside MØs. However, the mechanisms underlying this observation remain unclear.

In summary, we conclude that RecA is required for maintaining the survival of Mtb exposed to UV *in vitro* in both stationary and exponential growth phases. However, either HR or NHEJ is sufficient for the survival and propagation of Mtb inside MØs. ONOO^-^, but not •O_2_^-^ or NO themselves, produced by MØs effectively inhibit the survival of Mtb lacking both HR and NHEJ systems.

## Supporting Information

Table S1 (DOC)
