# Supplementary material for: Either Non-Homologous Ends Joining or Homologous Recombination Is Required to Repair Double-Strand Breaks in the Genome of Macrophage-Internalized Mycobacterium tuberculosis
Source: PLoS One. 2014 Mar 21;9(3):e92799. doi: 10.1371/journal.pone.0092799 (PMC3962454; doi:10.1371/journal.pone.0092799)
Supplement: Table S1 — (DOC) [file pone.0092799.s001.doc]

| **Plasmid** |  | **Description** | | | **Source** |
| --- | --- | --- | --- | --- | --- |
| pJET 1.2 |  | Blunt cloning vector, Amp^R^ | | | Fermentas |
| pMV306Km |  | Mycobacterial integrating vector, Kan^R^ | | | Med-Immune Inc. |
| p2NIL |  | Recombination vector, nonreplicating in mycobacteria, Kan^R^ | | | Parish T and Stoker NG, 2000 |
| pGoal17 |  | Source of PacI cassette, Amp^R^ | | | Parish T and Stoker NG, 2000 |
| pAB 215 |  | vector used for gene replacement with inactivated *ku,ligD*_Tb_ | | | This work |
| pAB 214 |  | *ku*_Tb_ PstI–HindIII fragment including 3’ end and its downstream region in p2NIL, Kan^R^ | | | This work |
| pAB 213 |  | *ligD*_Tb_ BamHI–HindIII fragment including 3’ end and its upstream region in pAB214, Kan^R^ | | | This work |
| pMG 22 |  | vector used for gene replacement with inactivated *recA*_Tb_ | | | This work |
| pMG 19 |  | *recA*_Tb_ PstI–HindIII fragment including 5’ end and its downstream region in p2NIL, Kan^R^ | | | This work |
| pMG 20 |  | *recA*_Tb_ BamHI–HindIII fragment including 3’ end and its upstream region in pAB, Kan^R^ | | | This work |
|  |  |  | | |  |
| pAB 219 |  | complementation vector (*ku,ligD*_Tb_ under natural promoter in pMV306, Km^R^) | | | This work |
| pMK 204 |  | complementation vector (*recA*_Ms_ under natural promoter in pMV306, Km^R^) | | | This work |
| **Strains** | | | | | **Source** |
| *M. smegamtis* mc^2^155 | | | | | Snapper SB et al., 1990 |
| *M. tuberculosis* H_37_ Rv | | | | | ATCC collection |
|  | | | | |  |
| *M. tuberculosis* Δ(*ku,lig*D) (mutant strain with inactivated *ku*_Tb_ *and ligD*_Tb_ genes) | | | | | This work |
|  | | | | |  |
| *M. tuberculosis* Δ *recA* (mutant strain with inactivated *recA*_Tb_) | | | | | This work |
|  | | | | |  |
| *M. tuberculosis* Δ(*ku,ligD,recA*) (mutant strain with inactivated *ku*_Tb_*, ligD*_Tb_, and *recA*_Tb_ genes) | | | | | This work |
|  | | | | |  |
| *M. tuberculosis* Δ(*ku,lig*D)- *ku,lig*D_Pown_ (mutant strain complemented with *ku* and *lig*D_Tb_ genes under natural promoter) | | | | | This work |
| *M. tuberculosis* Δ *recA-recA_MsPown_* (mutant strain complemented with *recA*_Ms_ under natural promoter ) | | | | | This work |
| **Primers used for gene replacement** | |  | **Primer name** | **Primer sequence** | |
| *GR1ku_Tb_* | |  | TbKu GR1-s | 5’ aactgcaggccgccaccatcgtgtcgc 3’ | |
| *GR2ku_Tb_*  *GR3ligD_Tb_*  *GR4ligD_Tb_*  *GR1recA_Tb_*  *GR2recA_Tb_*  *GR3recA_Tb_*  *GR4recA_Tb_* | |  | TbKu GR2-rev  TbLigD GR3-s  TbLigD GR4-rev  TBRecA GR1-s  TBRecA GR2-rev  TBRecA GR3-s  TBRecAGR4-rev | 5’ cgaagcttcgagaccgaccggatggcg 3’  5’ cgaagcttaatgcttgccacgcacggc 3’  5’ cgggatcctccagcatctgcaggtcccg 3’  5’ gcggactggctgcaggcacc 3’  5’ gcaagctttgcctgttccccggtgtccg 3’  5’ aaggaaaagcttggcattggtgc 3’  5’ aaggcatttggacgtgaactcgac 3’ | |
| **Primers used for complementation** | |  | **Primer name** | **Primer sequence** | |
| *recA1*  *recA2*  *ku-ligD1*  *ku-ligD2* | |  | MsrecAr  MsrecAPs  Ku-DTbXb  Ku-DTbHi | 5’gcgaattctccgacggctcagaagtcaacc3’  5’gctctagagatgaacaacagcgaaccggg3’  5’gctctagaggctgtcacggaggcgttggg3’  5’cgaagcttgcactcccgttcgttcgccg3’ | |

**1. Snapper SB, Melton RE, Mustafa S, Kieser T, Jacobs Jr. WR.** 1990. Isolation and characterization of efficient plasmid transformation mutants of *Mycobacterium smegmatis*. Mol. Microbiol. **4:**1911–1919.

**2. Parish T, Stoker NG.** 2000. Use of a flexible cassette method to generate a double unmarked *Mycobacterium tuberculosis* tlyA plcABC mutant by gene replacement. Microbiology. **146:**1969–1975.
